# Hypoxia Promotes Vascular Smooth Muscle Cell Proliferation through microRNA-Mediated Suppression of Cyclin-Dependent Kinase Inhibitors

**DOI:** 10.3390/cells8080802

**Published:** 2019-07-31

**Authors:** Jihui Lee, Hara Kang

**Affiliations:** Division of Life Sciences, College of Life Sciences and Bioengineering, Incheon National University, Incheon 406-772, Korea

**Keywords:** vascular smooth muscle cells, microRNAs, cyclin-dependent kinase inhibitors, hypoxia

## Abstract

Regulation of vascular smooth muscle cell (VSMC) proliferation is essential to maintain vascular homeostasis. Hypoxia induces abnormal proliferation of VSMCs and causes vascular proliferative disorders, such as pulmonary hypertension and atherosclerosis. As several cyclin/cyclin-dependent kinase (CDK) complexes and CDK inhibitors (CKIs) control cell proliferation, in this study, we investigated CKIs involved in the hypoxia-induced proliferation process of human primary pulmonary artery smooth muscle cells to understand the underlying molecular mechanism. We demonstrated that p15, p16, and p21 are downregulated in pulmonary artery smooth muscle cells when exposed to hypoxia. In addition, we identified novel hypoxia-induced microRNAs (hypoxamiRs) including miR-497, miR-1268a, and miR-665 that are upregulated under hypoxia and post-transcriptionally regulate *p15*, *p16*, and *p21* genes, respectively, by directly targeting their 3’UTRs. These miRNAs promoted the proliferation of VSMCs, and their inhibition decreased VSMC proliferation even in hypoxic conditions. Overall, this study revealed that miRNA-mediated regulatory mechanism of CKIs is essential for hypoxia-induced proliferation of VSMCs. These findings provide insights for a better understanding of the pathogenesis of vascular proliferative disorders.

## 1. Introduction

Hypoxia has been linked to the control of a wide range of cellular responses including cell proliferation, apoptosis, and inflammation [1]. In vascular smooth muscle cells (VSMCs), hypoxia stimulates abnormal cell proliferation and migration [2]. VSMC proliferation is an essential process that triggers the development of vascular remodeling, leading to the pathogenesis of many vascular proliferative disorders, including pulmonary hypertension and atherosclerosis [3]. A better understanding of the molecular mechanisms underlying VSMC proliferation in response to hypoxia would have valuable therapeutic implications.

Cell proliferation is controlled by multiple signaling pathways integrated into the basic cell cycle regulatory machinery by coordinating activities of cyclin/cyclin-dependent kinase (CDK) complexes [4]. CDK inhibitors (CKIs) restrict the activities of cyclin–CDK complexes and consequently restrain cell proliferation during development, differentiation, and response to cellular stresses [5]. In metazoans, two CKI gene families have been defined based on their evolutionary origins, structure, and CDK specificities. The inhibitors of CDK4 (INK4) gene family encodes *p15* (CDKN2B), *p16* (CDKN2A), *p18* (CDKN2C), and *p19* (CDKN2D), all of which bind to CDK4 and CDK6 and inhibit their kinase activities by interfering with their association with D-type cyclins [5]. The Cip/Kip family members encode *p21* (CDKN1A), *p27* (CDKN1B), and *p57* (CDKN1C), which bind to a wide range of G1/S and S-phase cyclin-CDK complexes and restrict their activities [4]. The CKIs have different patterns of expression in vascular diseases [6]. For example, *p21* and *p27* are upregulated during arterial repair and negatively regulate the proliferation of VSMCs [7]. However, the mechanism underlying CKI regulation in VSMCs upon hypoxia has been elusive.

MicroRNAs (miRNAs) are critical post-transcriptional regulators of gene expression and coordinate expression of a network of genes implicated in cell proliferation, apoptosis, and inflammation [8,9]. Notably, in response to hypoxia, miRNAs termed hypoxamiRs mediate hypoxia-related phenotype of VSMCs [10,11,12,13]. For example, the expression of miR-9, miR-214, and miR-322 are upregulated in response to hypoxia, and which, in turn, promote the proliferation of VSMCs [14,15,16,17]. However, the expression of miR-100, miR-103, and miR-107 are downregulated under hypoxia condition to promote hypoxia-induced VSMC proliferation [18]. Thus, we hypothesized that hypoxamiRs might control CKI expression and, consequently, regulate the miRNA–CKI axis during the hypoxia-induced proliferation of VSMCs.

In this study, we found that the expression of CKIs, such as *p15*, *p16*, and *p21*, was affected significantly in hypoxia-exposed VSMCs. We identified miRNAs, including miR-497, miR-1268a, and miR-665, which post-transcriptionally regulate those CKIs under hypoxia. Furthermore, we provide evidence for the critical role of these novel hypoxamiRs in the proliferation of VSMCs. Our findings expand the understanding of the molecular mechanism of the miRNA–CKI axis in hypoxia-induced VSMC proliferation.

## 2. Material and Methods

### 2.1. Cell Culture and Hypoxia

Human primary pulmonary artery smooth muscle cells (PASMCs) were purchased from Lonza (CC-2581) and were maintained in Sm-GM2 medium (Lonza, Basel, Switzerland) containing 5% fetal bovine serum (FBS). For hypoxia, the cells were placed in fresh medium and incubated in a sealed modular incubator chamber (Billups-rothenberg inc., San Diego, California, USA) for 24 h at 37 °C after flushing with a mixture of 5% CO_2_, 1% O_2_, and 94% N_2_ for 4 min.

### 2.2. Quantitative Reverse Transcriptase-PCR (qRT-PCR)

Quantitative analysis of the change in expression levels was performed using real-time PCR. The mRNA levels were normalized to 18S rRNA. The primers used were as follows:

*p115*, 5′-CGCCCACAACGATTTATTT-3′ and 5′-TTCGCTTCATGGTGAGTGTC-3′;
*p16*, 5′-ATATGCCTTCCCCCACTACC-3′ and 5′-CCCCTGAGCTTCCCTAGTTC-3′;
*p18*, 5′-ACGTCAATGCACAAAATGGA-3′ and 5′-TCAGCTTGAAACTCCAGCAA-3′;
*p19*, 5′-TGCAGGTCATGATGTTTGG-3′ and 5′-CAGCAGTGTGACCCTCTTGA-3′;
*p21*, 5′-GACACCACTGGAGGGTGACT-3′ and 5′-CCACATGGTCTTCCTCTGCT-3′;
*p27*, 5′-GAGTGGCAAGAGGTGGAGAA-3′ and 5′-GCGTGTCCTCAGAGTTAGCC-3′;
*p57*, 5′-CAGGAGCCTCTCGCTGAC-3′ and 5′-CTTCTCAGGCGCTGATCTCT-3′.

For quantification of mature miRNAs such as miR-497, miR-1268a, and miR-665, the miScript PCR assay kit (Qiagen, Hilden, Germany) was used according to the manufacturer’s instructions. Data analysis was performed using a comparative C_T_ method in the Bio-Rad software. miRNA levels were normalized to U6 small nuclear RNA. Three experiments were performed in triplicate, and the average results with standard errors are presented.

### 2.3. miRNA Mimics and Anti-miRNA Oligonucleotides

Chemically modified double-stranded RNAs designed to mimic the endogenous mature miR-497, miR-1268a, miR-665, miR-410-3p, miR-132-3p, and miR-212-3p were purchased from Genolution Pharmaceuticals (Seoul, Republic of Korea). Antisense inhibitor RNAs (anti-miR-497, anti-miR-1268a, and anti-miR-665) and negative control miRNA were purchased from Bioneer (Daejeon, Republic of Korea). The miRNA mimics and anti-miRNA oligonucleotides were transfected at 5 nM and 50 nM, respectively, using RNAi Max (Invitrogen, Carlsbad, California, CA, USA) according to the manufacturer’s protocol.

### 2.4. Luciferase Reporter Constructs

The 3’UTR sequence of *p15*, *p16*, and *p21* including the predicted miRNA recognition element (MRE) were cloned into the pIS0 vector (Addgene, Watertown, Massachusetts, MA, USA) containing the luciferase gene. RT-PCR was used to amplify the 3’UTR sequence of *p15*, *p16*, and *p21* from mRNA isolated from PASMCs. The primers used were as follows:

*p15* 3′UTR, 5′-AGAGAGCTCAGTGGAGAAGGTGCGACAG-3′ and 5′-AGAGGCCGGCCAGTAGCAAGTCATAAGGGGATTTC-3′;

*p16* 3′UTR, 5′-AGAGAGCTCCGATTGAAAGAACCAGAGAGG-3′ and 5′-AGAGGCCGGCCGAGCTTTGGTTCTGCCATTT-3′; 

*p21* 3′UTR, 5′-TACGAGCTCTAATCCGCCCACAGGAAG-3′ and 5′-TTCGGCCGGCCTTTGATGATGCCCCCACT-3′.

The putative MRE-deleted mutant constructs for *p15* and *p21* were generated using the pIS0 vector. The RT-PCR primers used for mutant constructs were as follows:

*p15* mutant, 5′-CATGAGCTCCCACAACGATTTATTTTCTTACC-3′ and 5′-AGAGGCCGGCCAGTAGCAAGTCATAAGGGGATTTC-3′;

*p21* mutant, 5′-TACGAGCTCCCTCATGGCCCCTCTGAC-3′ and 5’-TCAGGCCGGCCGTTTACAGTCTAGGTGGAGAAACG-3’.

For the putative MRE sequence-mutated construct for p16, an upstream region and a downstream region of the MRE site in the 3’UTR were amplified using primers including *Xho*I enzyme recognition sequences. Two PCR products were digested by *Xho*I enzyme, ligated to change the MRE sequence into the *Xho*I recognition sequence and then cloned into the pIS0 vector. 5′-AGAGAGCTCCGATTGAAAGAACCAGAGAGG-3′ and 5′-ACACTCGAGGAGTGCTCACTCCAGAAAAC-3′ were used for the amplification of the upstream region. 5′-ACTCTCGAGTAAGCGCACATTCATGTGGG-3′ and 5′-AGAGGCCGGCCGAGCTTTGGTTCTGCCATTT-3′ were used for the amplification of the downstream region.

### 2.5. Luciferase Assay

Cos7 cells were cotransfected with 5 nM miR-497, miR-1268a, miR-665, or control mimic and luciferase reporter constructs using Lipofectamine 2000 (Life technologies, Carlsbad, California, CA, USA). A β-galactosidase expression plasmid was used as an internal transfection control. Twenty-four hours later, luciferase assays were performed, and luciferase activity was presented after normalization to β-galactosidase activity.

### 2.6. Immunoblotting

Cells were lysed in TNE buffer (50 mM Tris–HCl (pH 7.4), 100 mM NaCl, and 0.1 mM EDTA) and total cell lysates were separated by SDS-PAGE, transferred to PVDF membranes, immunoblotted with antibodies and visualized using an enhanced chemiluminescence detection system (Amersham Biosciences, Little Chalfont, UK). The antibodies against *p15* (sc-612), *p21* (sc-397), and β-actin (sc-47778) were purchased from Santa Cruz (Dallas, Texas, TX, USA). An anti-p16 antibody (#554079) was purchased from BD Biosciences (San Jose, California, CA, USA).

### 2.7. Cell Proliferation Assay

CellTiter-Glo® Luminescent Cell Viability Assay (Promega, Madison, Wisconsin, WI, USA) was used to determine the number of viable cells in culture. Briefly, 5 × 10^3^ cell/well were seeded in 96-well plates in triplicate. After transfection of miRNAs for 3 days, a volume of CellTiter-Glo reagent (Promega, Madision, Wisconsin, WI, USA) equal to the volume of cell culture medium was added to each well. The plates were shaken for 2 min to induce cell lysis and further incubated for 10 min to stabilize luminescent signal. Cell proliferation was measured by reading the absorbance at 490 nm using a GloMax 96 Microplate Luminometer (Promega, Madision, Wisconsin, WI, USA).

### 2.8. Immunofluorescence Staining

Equal amounts of PASMCs were seeded in chamber well slides and transfected with control mimic, miR-497, miR-1268a, miR-665, anti-miR-497, anti-miR-1268a, or anti-miR-665. Cells were then exposed to normoxia or hypoxia and fixed in 2% paraformaldehyde. The slides were blocked in 3% BSA in PBS and permeabilized in 0.1% Triton X-100 (Sigma-Aldrich, Missouri, MO, USA) in PBS. Probing was done with rabbit anti-human Ki-67 antibody (Abcam, #ab16667) and goat anti-rabbit IgG (H+L) cross-adsorbed secondary antibody, Alexa Fluor 488 (Thermo Fisher Scientific, #A-11008). Nuclei were stained with Hoechst 33342 (Thermo Fisher Scientific, #62249). The slides were imaged by a Zeiss Axio Imager Z1 microscope (Oberkochen, Germany). At least 2000 cells were counted per condition, and the percentages of Ki-67 positive cells were presented. The results are the mean ± S.E. for triplicate assays.

### 2.9. Statistical Analysis

For each of the assays, three experiments were performed in triplicate, and the results were presented as the average with standard error. Statistical analyses were performed by an analysis of variance followed by Student’s *t* test using Prism 8 software (GraphPAD Software Inc., San Diego, CA, USA). *p* values of <0.05 were considered significant and are indicated with asterisks.

## 3. Results

### 3.1. Hypoxia Downregulates Expression Levels of Specific CDK Inhibitors

**Figure 1:**
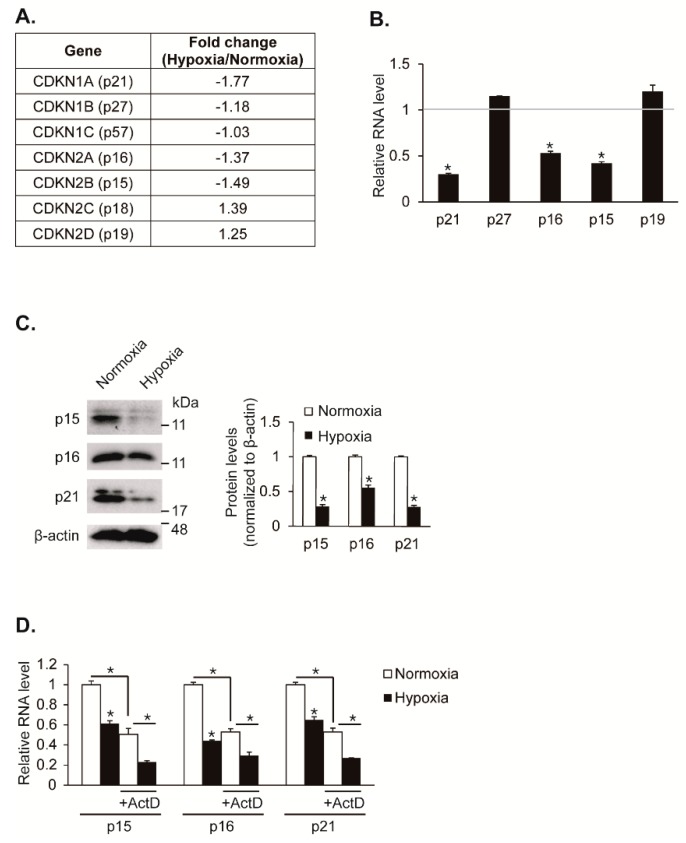
The expression of cyclin-dependent kinase (CDK) inhibitors, p15, p16, and p21, are downregulated under hypoxia. (**A**) Expression levels of CDK inhibitors were compared in PASMCs exposed to normoxia or hypoxia by next generation sequencing (NGS)-based RNA sequencing. (**B**) mRNA levels of CDK inhibitors normalized to 18S rRNA were examined by qRT-PCR in PASMCs exposed to normoxia or hypoxia for 24 h. Data represent the mean ± S.E. of triplicates. * *p* < 0.05. (**C**) Total cell lysates from pulmonary artery smooth muscle cells (PASMCs) exposed to normoxia or hypoxia for 24 h were subjected to immunoblot analysis with antibodies against p15, p16, p21, or β-actin. By densitometry, relative amounts of indicated proteins normalized to β-actin were quantitated. * *p* < 0.05. (**D**) PASMCs were exposed to normoxia or hypoxia in the absence or presence of actinomycin D and their transcript levels of p15, p16, and p21 normalized to 18S rRNA were examined by qRT-PCR. * *p* < 0.05.

Cell proliferation is regulated by hypoxia. Particularly, hypoxia promotes proliferation in VSMCs. We have previously analyzed small non-coding RNAs and coding transcripts using next-generation sequencing (NGS)-based RNA sequencing in hypoxic pulmonary artery smooth muscle cells (PASMCs) to elucidate the molecular mechanism of hypoxia-induced VSMC proliferation in depth [12]. We hypothesized that CDK inhibitors are regulated by hypoxia and responsible for induced VSMC proliferation. Indeed, we observed that specific CDK inhibitors such as CDKN1A (p21), CDKN2A (*p16*), and CDKN2B (*p15*) are downregulated under hypoxia in our NGS-based RNA sequencing data (Figure 1A). To validate that hypoxia downregulates expression levels of CDK inhibitors, PASMCs were exposed to hypoxia for 24 h and then analyzed for gene expression using a qRT-PCR approach (Figure 1B). Consistent with the NGS-based RNA sequencing data, the mRNA levels of *p15*, *p16*, and *p21* genes were reduced by approximately 58%, 47%, and 70%, respectively, upon exposure to hypoxia. The mRNA levels of *p19* and *p27* genes were not significantly changed under hypoxia. The levels of *p18* and *p57* transcripts were not detectable due to their very low expression in PASMCs. Consistent with gene expression results, immunoblot analyses showed that the levels of p15, p16, and p21 proteins decreased in a hypoxic condition (Figure 1C). Taken together, our findings suggest that expression levels of CDK inhibitors such as p15, p16, and p21 are reduced in PASMCs by hypoxia.

To test whether the decrease in these CKI levels under hypoxia is due to their post-transcriptional regulation, PASMCs were treated with the RNA polymerase II inhibitor actinomycin D (ActD) to block the synthesis of new transcripts prior to an exposure to hypoxia, followed by measuring the levels of *p15*, *p16*, and *p21* transcripts using a qRT-PCR method (Figure 1D). In the absence of actinomycin D, the CKI transcript levels were decreased following hypoxia exposure for 24 h. Upon actinomycin D treatment, the levels of *p15*, *p16*, and *p21* transcripts were reduced by approximately 50%, and the hypoxia-mediated inhibition still occurred, which suggests that existing CKI transcripts were downregulated by hypoxia. Therefore, CKI expressions are likely to be downregulated post-transcriptionally under hypoxia conditions.

### 3.2. Identification of miRNAs That Target CDK Inhibitors under Hypoxia

**Figure 2:**
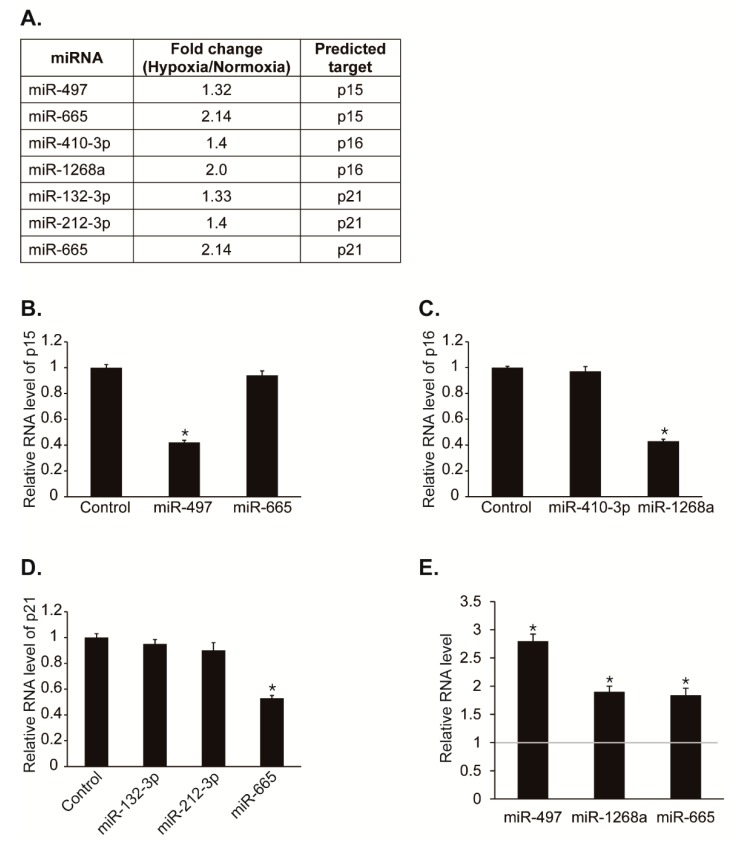
Hypoxia-induced miRNA candidates that regulate CDK inhibitors. (**A**) Expression levels of miRNAs were compared in PASMCs exposed to normoxia or hypoxia by NGS-based small RNA sequencing. (**B)** PASMCs were transfected with control mimic, miR-497, or miR-665 for 24 h and subjected to qRT-PCR analysis of *p15*. The relative levels of mRNA expression normalized to 18S rRNA were quantitated. Data represent the mean ± S.E. of triplicates. * *p*< 0.05. (**C**) Levels of endogenous *p16* mRNA relative to 18S rRNA were quantified by qRT-PCR analysis in PASMCs transfected with control mimic, miR-410-3p, or miR-1268a. * *p* < 0.05. (**D**) Levels of *p21* mRNA relative to 18S rRNA were quantified by qRT-PCR analysis in PASMCs transfected with control mimic, miR-132-3p, miR-212-3p, or miR-665. * *p* < 0.05. (**E**) Expression levels of miR-497, miR-1268a, and miR-665 normalized to U6 snRNA were examined by qRT-PCR in PASMCs exposed to normoxia or hypoxia for 24 h. * *p* < 0.05.

To test our hypothesis that miRNAs regulates CDK inhibitors post-transcriptionally to promote cell proliferation under hypoxia, we first searched for miRNAs predicted to target potential miRNA recognition elements (MREs) within the 3′UTR of *p15*, *p16*, or *p21* using computer algorithms such as TargetScan, miRWalk, miRanda, and RNA22. If the expression of CDK inhibitors is repressed post-transcriptionally by miRNAs under hypoxia, then the miRNAs might be upregulated by hypoxia. Thus, we observed whether the predicted miRNAs are upregulated under hypoxia in our NGS-based small RNA sequencing data with hypoxic PASMCs (Figure 2A) [12]. Among miRNAs predicted to target *p15*, miR-497 and miR-665 levels were increased by hypoxia approximately 1.3-fold and 2.1-fold, respectively. Among miRNAs predicted to target *p16*, miR-410-3p and miR-1268a levels were increased by hypoxia approximately 1.4-fold and 2-fold, respectively. Among miRNAs predicted to target *p21*, miR-132-3p, miR-212-3p, and miR-665 levels were increased by hypoxia approximately 1.3-fold, 1.4-fold and 2.1-fold, respectively. These results suggest that hypoxia-induced miRNAs such as miR-497, miR-665, miR-410-3p, miR-1268a, miR-132-3p, or miR-212-3p might regulate the expression of CDK inhibitors.

To determine whether these miRNAs affect the expression of CDK inhibitors, we examined the levels of *p15*, *p16*, or *p21* after transfection of PASMCs with each miRNAs (Figure 2B–D). Expression levels of *p15*, *p16*, and *p21* were reduced by miR-497, miR-1268a, and miR-665, respectively. No other miRNAs have a significant effect on the expression of *p15*, *p16*, or *p21*.

We then validated that the expression of miR-497, miR-1268a, and miR-665 was induced in hypoxia using qRT-PCR (Figure 2E). The levels of miR-497, miR-1268a, and miR-665 were increased to approximately 2.8-fold, 1.9-fold, and 1.8-fold, respectively, after exposure to hypoxia for 24 h. Therefore, CDK inhibitors, p15, p16, and p21 might be regulated by hypoxia-induced miRNAs including, miR-497, miR-1268a, and miR-665.

### 3.3. Hypoxia-Induced miRNAs Regulates the Expression of CDK Inhibitors

**Figure 3:**
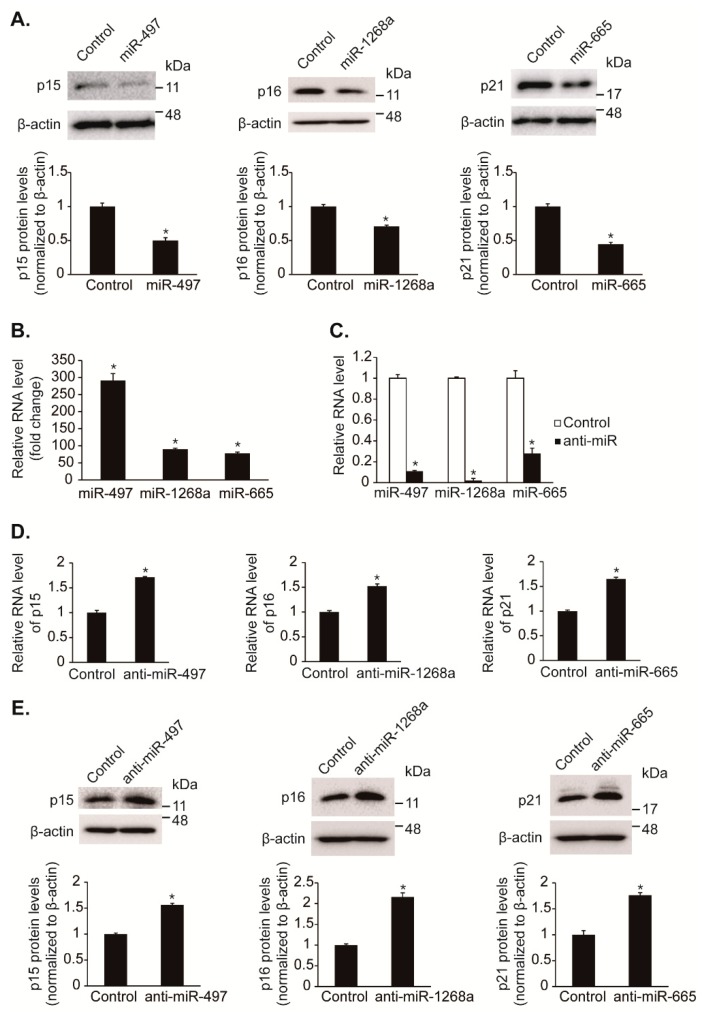
Identification of miRNAs that regulate endogenous levels of CDK inhibitors. (**A**) Total cell lysates from PASMCs transfected with control mimic, miR-497, miR-1268a, or miR-665 for 24 h were subjected to immunoblot analysis with antibodies against p15, p16, p21, or β-actin. By densitometry, relative amounts of CDK inhibitor (CKI) proteins normalized to β-actin were quantitated. * *p* < 0.05. (**B**,**C**) Expression levels of miR-497, miR-1268a, and miR-665 normalized to U6 snRNA were examined by qRT-PCR in PASMCs transfected with control mimic, miR-497, miR-1268a, miR-665, anti-miR-497, anti-miR-1268a, or anti-miR-665. * *p* < 0.05. (**D**) Levels of endogenous *p15*, *p16*, or *p21* mRNAs relative to 18S rRNA were quantified by qRT-PCR analysis in PASMCs transfected with control mimic, anti-miR-497, anti-miR-1268a, or anti-miR-665. * *p* < 0.05. (**E**) Total cell lysates from PASMCs transfected with control mimic, anti-miR-497, anti-miR-1268a, or anti-miR-665 were subjected to immunoblot analysis with antibody against p15, p16, p21, or β-actin. By densitometry, relative amounts of CKI proteins normalized to β-actin were quantitated. * *p* < 0.05.

We further examined that hypoxia-induced miRNAs, such as miR-497, miR-1268a, and miR-665, reduce protein levels of CDK inhibitors, *p15*, *p16*, and *p21*. The endogenous protein levels of *p15*, *p16*, and *p21* in PASMCs were reduced to approximately 50%, 70%, and 44% by exogenous miR-497, miR-1268a, and miR-665 mimics, respectively, compared with control (Figure 3A). The overexpression of miRNAs was ascertained by qRT-PCR (Figure 3B).

To assess whether endogenous miR-497, miR-1268a, and miR-665 regulate the expression of p15, p16, and p21, these miRNAs were inhibited by transfection of antisense RNA oligonucleotides complementary to the miRNA sequences (anti-miR-497, anti-miR-1268a, and anti-miR-665) in PASMCs. Anti-miR-497, anti-miR-1268a, and anti-miR-665 reduced the level of the respective miRNAs to approximately 11%, 2%, and 28% of the basal level (Figure 3C). We observed that the levels of *p15*, *p16*, and *p21* mRNAs were elevated approximately 1.7-fold, 1.5-fold, and 1.65-fold by anti-miR-497, anti-miR-1268a, and anti-miR-665, respectively (Figure 3D). Consistent with mRNA levels, protein levels of p15, p16, and p21 were elevated approximately 1.56-fold, 2.16-fold, and 1.76-fold upon transfection with anti-miR-497, anti-miR-1268a, and anti-miR-665, respectively (Figure 3E). These results indicate that hypoxia-induced miRNAs, miR-497, miR-1268a, and miR-665, regulate expression of the CDK inhibitors, p15, p16, and p21, respectively.

### 3.4. Validation of CDK Inhibitors as Novel Targets of miR-497, miR-1268a, and miR-665

**Figure 4:**
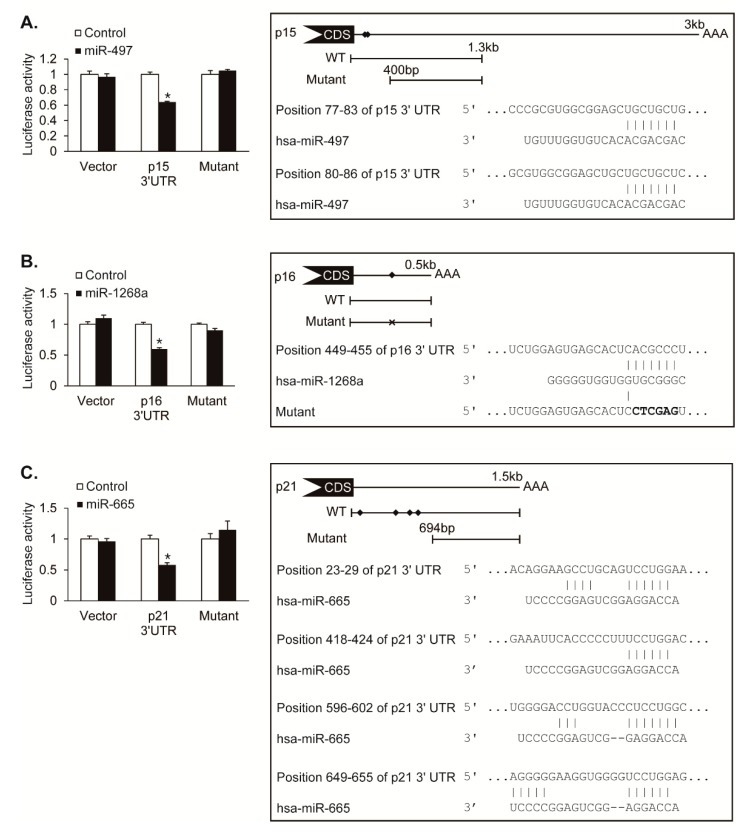
Hypoxia-induced miRNAs target CDK inhibitors. (**A**–**C**) (**Left panel**) Luciferase activity of constructs with the 3′UTR of *p15*, *p16*, or *p21* including the wild-type miRNA MREs, the mutant 3′UTR deleted miRNA recognition element (MRE) for miR-497 or miR-665, or the mutant 3′UTR with mutated MRE sequence for miR-1268a were examined in Cos7 cells by transfecting control, miR-497, miR-1268a, or miR-665 mimic. A luciferase vector without 3′UTR sequence (Vector) was used as a negative control. Data represent the mean ± S.E. of triplicates. * *p* < 0.05. (**Right panel**) Schematic diagrams of the predicted MREs of miR-497, miR-1268a, and miR-665 in the 3′UTR of *p15*, *p16* and *p21* transcripts, and the luciferase reporter constructs used for luciferase assays. The predicted MREs are marked with diamonds. CDS and AAA stand for protein coding sequence and poly(A) tail, respectively. Mutations introduced in the MRE to disrupt a base pairing with miR-1268a sequence are indicated as X. Sequences of the 3′UTR of CDK inhibitors and the predicted MRE of miRNAs are shown. Perfect base matches are indicated by a line.

To determine whether CDK inhibitors, p15, p16, and p21, are direct targets of miR-497, miR-1268a, and miR-665, we performed the luciferase assay using luciferase reporter constructs containing the 3′UTR of *p15*, *p16*, and *p21*. We also generated constructs with mutations in the putative MRE sequences for the luciferase assay. The luciferase activity of the *p15* 3′UTR construct was reduced by approximately 36% upon cotransfection with miR-497, whereas the activity of the putative MRE-deleted mutant construct was not affected by miR-497, demonstrating that miR-497 directly targets 3′UTR of *p15* transcript (Figure 4A). The luciferase activity of the *p16* 3′UTR construct was reduced by approximately 40% by miR-1268a, whereas the activity of the putative MRE sequence-mutated construct was not changed by miR-1268, suggesting that miR-1268a directly targets the MRE sequence within 3′UTR of the *p16* transcript (Figure 4B). The luciferase activity of the *p21* 3′UTR construct was reduced by approximately 42% by miR-665, whereas the activity of the putative MRE-deleted mutant construct was not affected by miR-665, suggesting that miR-665 regulates the *p21* expression through targeting its 3′UTR (Figure 4C). These results indicate that miR-497, miR-1268a, and miR-665 downregulate their novel targets, *p15*, *p16*, and *p21* genes, respectively, by direct binding to 3′UTR of their target mRNAs.

### 3.5. miR-497, miR-1268a, and miR-665 Regulate VSMC Proliferation

**Figure 5:**
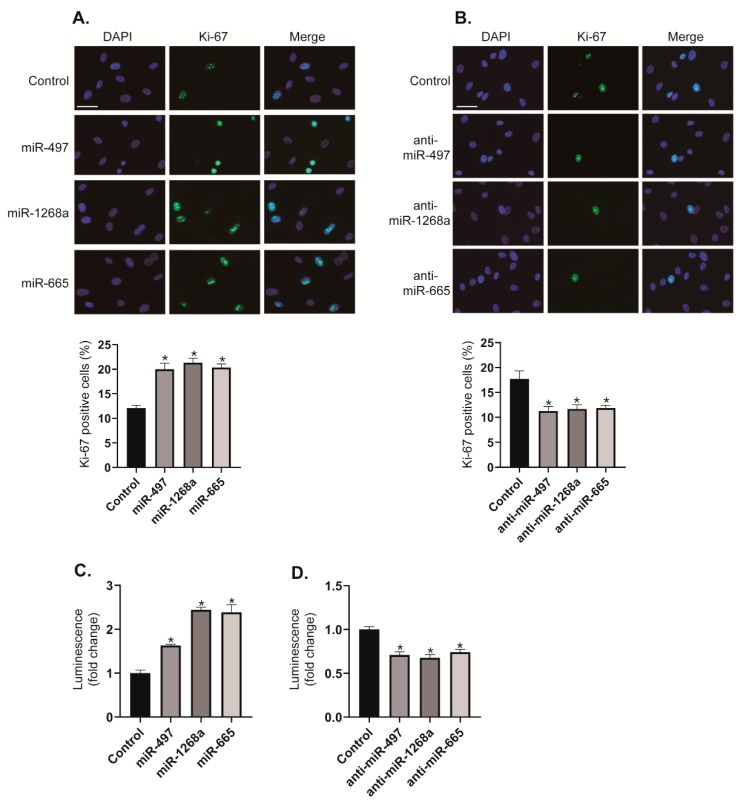
miR-497, miR-1268a, or miR-665 promotes the proliferation of vascular smooth muscle cells (VSMCs). (**A**,**B**) Representative images of Ki-67 immunostaining of PASMCs transfected with control, miR-497, miR-1268a, miR-665, anti-miR-497, anti-miR-1268a, or anti-miR-665, and calculation of Ki-67 index. Approximately, 200 cells from at least 10 independent fields were counted for each condition, and Ki-67 positive cells are presented as a percentage of the total population. Scale bar represents 20 μ. * *p* < 0.05. (**C**,**D**) Luminescent signals for viable PASMCs transfected with control, miR-497, miR-1268a, miR-665, anti-miR-497, anti-miR-1268a, or anti-miR-665 for 3 days. * *p* < 0.01.

We investigated the effects of the miR-497, miR-1268a, and miR-665 on VSMC proliferation by immunostaining with an antibody against Ki-67. When PASMCs were transfected with miR-497, miR-1268a, or miR-665 mimics, the number of Ki-67 positive proliferating cells increased to approximately 1.67-fold, 1.78-fold, and 1.7-fold, respectively, compared with control cells (Figure 5A). In contrast, when activities of miR-497, miR-1268a, or miR-665 were downregulated by transfection with anti-miR-497, anti-miR-1268a, or anti-miR-665, the number of Ki-67 positive proliferating cells decreased by approximately 36%, 34%, and 33%, respectively, compared with control cells (Figure 5B). We also performed CellTiter-Glo® Luminescent Cell Viability Assay to measure ATP as an indicator of the number of viable cells [19]. Because the level of ATP is directly proportional to the number of cells, this assay has been used for cell proliferation assay. Consistent with the results of Ki-67 immunostaining, the proliferation of PASMCs is promoted by transfecting these cells with miR-497, miR-1268a, or miR-665 mimics (Figure 5C), whereas the rate of proliferation is reduced by transfecting these cells with anti-miRNAs (Figure 5D). These results imply that miR-497, miR-1268a, and miR-665 function as pro-proliferative regulators in VSMCs.

### 3.6. Modulation of miRNAs Regulates the Hypoxia-Induced Proliferation of VSMCs

**Figure 6:**
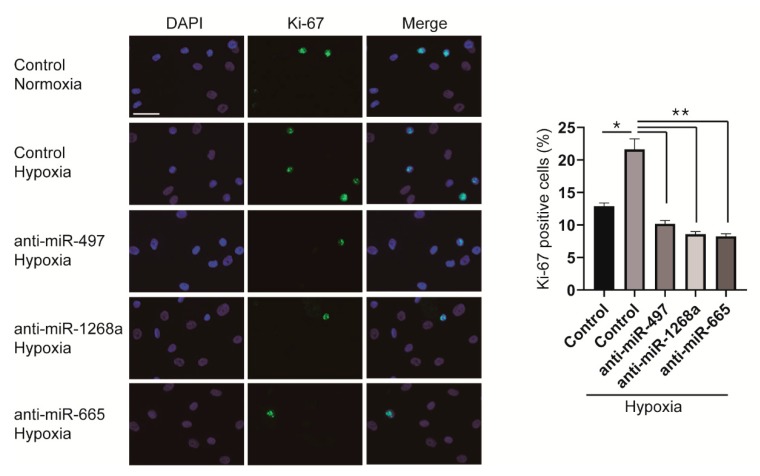
Inhibition of miR-497, miR-1268a, or miR-665 prevents the hypoxia-induced proliferation of VSMCs. (**Left panel**) Representative images of Ki-67 immunostaining of control, anti-miR-497, anti-miR-1268a, or anti-miR-665-transfected PASMCs after exposure to normoxia or hypoxia for 24 h. Scale bar represents 20 μm. (**Right panel**) Calculation of Ki-67 index. * *p* < 0.001; ** *p* < 0.0001.

Since miR-497, miR-1268a, and miR-665 promote the proliferation of VSMCs, we examined whether the hypoxia-induced proliferative response of VSMCs is prevented by downregulating those miRNAs that we have identified. PASMCs were transfected with anti-miRNAs 6 h before hypoxia exposure and stained with an antibody against Ki-67. In control mimic-transfected cells, the number of proliferating cells increased to approximately 1.7-fold after exposure to hypoxia for 24 h; however, the number of Ki-67 positive cells transfected with anti-miRNAs did not increase even after hypoxia exposure (Figure 6). It indicates that downregulation of miRNAs, such as miR-497, miR-1268a, and miR-665, by their respective anti-miRNAs inhibits hypoxia-induced proliferation of VSMCs.

## 4. Discussion

Vascular proliferative disorders, including pulmonary hypertension, are characterized by enhanced proliferation and suppressed apoptosis of VSMCs [3]. Under hypoxic conditions, the proliferation of VSMCs is enhanced, resulting in vasoconstriction of the pulmonary vasculature in concert with vascular remodeling, which finally leads to pulmonary hypertension [2]. CKIs regulate cell cycle progression in VSMCs and thus, appear to be of significant importance in mediating the remodeling of pulmonary arteries in response to hypoxia [6]. Therefore, the detailed regulatory mechanism of CKIs, which serve as endogenous inhibitors of VSMC proliferation, is essential for the development of the potential therapies for vascular proliferative diseases. Since miRNAs are critical post-transcriptional regulators of gene expression and key mediators of the pathogenesis of vascular proliferative diseases, in this study, we first observed the downregulation of CKIs, including p15, p16, and p21, in primary cultured human PASMCs under the hypoxic condition and then characterized the post-transcriptional regulation of these CKIs by miRNAs. We identified miR-497, miR-1268a, and miR-665 as novel hypoxamiRs and discovered their novel functions in the regulation of VSMC proliferation by directly modulating the expression of p15, p16, or p21. Furthermore, we showed that the miRNA-mediated regulation of CKIs is essential for hypoxia-induced proliferation of VSMCs.

Although functions of miR-497, miR-665, and miR-1268a in the regulation of cellular responses to hypoxia have not yet been demonstrated and little is known about miR-1268a function, there is likely a clue for the link between these miRNAs and vascular proliferative diseases. For example, miR-665 was found significantly upregulated in heart and plasma during heart failure [20]. Moreover, miR-665 regulates the osteosarcoma cell proliferation, epithelial–mesenchymal transition (EMT) and invasion, implying that miR-665 might have a role in the pathogenesis of vascular proliferative diseases [21]. Aberrant expression of miR-497 has been frequently reported in cancer studies [22]. miR-497 was downregulated in certain types of cancer, including breast, gastric, endometrial, colorectal, and bladder cancer [23,24,25,26,27]. The enforced expression of miR-497 inhibited cell proliferation, migration, and invasion, implying that miR-497 functions as a tumor suppressor [22]. Although miR-497 mainly suppresses tumors, it also acts as an oncogene in other cancers [28,29]. For example, miR-497 enhances metastasis of oral squamous cell carcinoma through SMAD7 suppression [28]. In this study, we illustrated that miR-665, miR-497, and miR-1268a repress the expression of CKIs and consequently promote VSMC proliferation in hypoxic conditions.

CKIs are highly conserved cell cycle regulators and tumor suppressor genes implicated in the pathogenesis of several malignancies [4]. An imbalance of cell proliferation versus cell death and the cellular and molecular features observed in hypoxic pulmonary hypertension pathology resemble hallmark characteristics described for cancer. Indeed, p15 has been recently reported to be implicated as a candidate gene, which may be responsible, in part, for genetic cardiovascular disease [30]. Moreover, loss of p15 may not only promote cardiovascular disease through the development of atherosclerosis but may also impair TGF-β signaling and hypoxic neovessel maturation [30]. These recent studies make it plausible that the function of p15 is involved in hypoxia-induced VSMC proliferation. In this study, we demonstrated that p15 is downregulated under hypoxia condition so that VSMC proliferation is enhanced.

Regulation of p16 under hypoxia has been elucidated in mesenchymal stem cells (MSCs) [31]. The hypoxic condition maintains MSCs in an undifferentiated and senescence-free state by downregulating p16 [31]. In addition, p16 was decreased when pulmonary artery endothelial cell proliferation is induced by miR-215a [32]. However, miR-125a could neither induce proliferation nor negatively regulate p16 expression in PASMCs [32], implying there is another miRNA which regulated *p16* in PASMCs. In this study, we observed that the expression of *p16* is modulated by miR-1268a upon hypoxia in VSMCs, and consequently, cell proliferation is regulated under hypoxia.

It is well known that regulation of p21 is essential for VSMC proliferation [33,34]. p21 is upregulated in arteries after vascular injury and the overexpression of p21 in VSMCs results in inhibition of cell growth [35]. Several miRNAs regulating expression of *p21* have been identified [36]. miR-130a controls VSMC proliferation by directly targeting *p21*. miR-130a enhances hypoxia-induced proliferation of VSMCs and might be involved in the development of right ventricular hypertrophy and vascular remodeling in pulmonary hypertension [36,37]. In addition, it was demonstrated that miR-17 regulates the expression of *p21*, and the inhibition of miR-17 improves heart and lung function in experimental pulmonary hypertension by interfering with lung vascular and right ventricular remodeling [38]. We examined whether these miRNAs are downregulated in hypoxia-exposed PASMCs. From our NGS-based small RNA sequencing data, the level of miR-17 expression was not changed in response to hypoxia, and the expression of miR-130a was not determined [12]. It suggests that it is unlikely that miR-17 and miR-130a are responsible for the promotion of VSMC proliferation under hypoxia. In this study, we identified another miRNA, miR-665, that regulates the expression of *p21* and characterized its pro-proliferative function in VSMCs under hypoxia.

Primary cultured cells were isolated directly from the tissue and their characteristics were similar to that of the tissue, but they could have a limited ability to develop complex biological responses of in vivo models. Further in vivo study will provide more compelling evidence of a potential therapeutic strategy to effectively control the development of vascular diseases.

## 5. Conclusions

A few studies suggest that CKIs are involved in the proliferation of VSMCs, but the regulation of CKIs by miRNAs associated with the hypoxia-induced proliferation of VSMCs has not been elucidated yet. We comprehensively examined the expression levels of CKIs in primary cultured human PASMCs under hypoxia and found that expression of p15, p16, and p21 was downregulated by hypoxia. The downregulation of CKIs is regulated by miRNAs induced by hypoxia, such as miR-497, miR-1268a, and miR-665. This hypoxia-induced miRNA-CKI axis is essential for the pro-proliferative function in VSMCs. Our understanding of the post-transcriptional regulation of CKIs in VSMC proliferation under hypoxia provides new insight into the mechanisms of vascular proliferative disorders.
